# Beta-Globin (HBB) Mutations and Catalase Gene Polymorphisms in Beta-Thalassemia Major Patients in Al-Diwaniyah, Iraq

**DOI:** 10.3390/genes17080906

**Published:** 2026-07-31

**Authors:** Huda Ahmed Fairooz, Rania Abdelhedi, Sarab Hussain Khalil, Najla Kharrat, Mounira Hmani

**Affiliations:** 1 Laboratory of Molecular and Functional Genetics, Faculty of Sciences of Sfax, Route de la Soukra Km 4, P.O. Box 1171, Sfax 3000, Tunisia; huda.fairooz@qu.edu.iq; 2 Medicine College, Al-Qadysiah University, Al-Diwaniyah 58001, Iraq; 3 Laboratory of Molecular and Cellular Screening Processes, Centre of Biotechnology of Sfax, University of Sfax, Sidi Mansour Road Km 6, P.O. Box 1177, Sfax 3018, Tunisia; rania.abdelhedi@cbs.rnrt.tn (R.A.); najla.kharrat@cbs.rnrt.tn (N.K.); 4 Human Anatomy Branch, College of Medicine, Ibn Sina University of Medical and Pharmaceutical Sciences, Baghdad 10012, Iraq; sarab.khaleel@ibnsina.edu.iq

**Keywords:** β-thalassemia major, *HBB* mutations, *CAT* polymorphisms, biochemical parameters oxidative stress, *HBB-CAT* interaction, risk stratification, Iraq

## Abstract

**Background/Objectives**: β-thalassemia major is a common inherited hemoglobin disorder caused by Hemoglobin Subunit Beta (*HBB*) mutations and often complicated by iron overload and oxidative stress. This study characterized the clinical, hematological, biochemical, and molecular profile of β-thalassemia major in Al-Diwaniyah, Iraq, focusing on *HBB* mutations and *CAT* promoter polymorphisms as oxidative modifiers. **Methods**: A retrospective case–control study included 100 transfusion-dependent patients and 50 healthy controls. Sociodemographic data, complete blood count, ferritin, lipid profile, catalase (CAT), and malondialdehyde (MDA) were assessed. *HBB* mutations, namely IVSI-1 (G>A), IVSI-6 (T>C), and CD39 (C>T), and *CAT* polymorphisms, namely rs1001179 (C>T) and rs7943316 (A>T), were genotyped. **Results**: Patients had high rates of family history (80%), consanguinity (80%), rural residence (74%), low education (60.6%), and poor socioeconomic status (71.4%). Compared with controls, they showed lower RBC, HGB, HCT, HDL, LDL, and CAT but higher ferritin, triglycerides, and MDA (all *p* < 0.001). Overall, 69% of patients carried at least one studied HBB mutations, while 31% remained uncharacterized. Mutant allele frequencies were 29% for IVSI-1 (A) and 25.5% each for IVSI-6 (C) and CD39 (T). The *CAT* polymorphism rs1001179 (C>T) was associated with disease risk (CT: 33% vs. 14%; OR = 3.25, *p* = 0.019), whereas rs7943316 (A>T) was not. IVS-I-1 (G>A)/CD39 (C>T) β^0^ mutations were associated with lower CAT activity (β = −0.205; *p* = 0.046). **Conclusions**: β-thalassemia in Al-Diwaniyah, Iraq, shows substantial clinical, genetic, and oxidative heterogeneity. Integrated HBB–CAT analysis may improve molecular diagnosis, risk stratification, and preventive strategies in high-consanguinity populations.

## 1. Introduction

β-Thalassemia is an autosomal recessive hemoglobinopathy (OMIM: 141900) caused by mutations in the *HBB* gene on chromosome 11 that result in reduced (β^+^) or absent (β^0^) synthesis of the β-globin chain, leading to excess unpaired α-chas that precipitate within erythroid precursors [1,2]. These free α-chains cause ineffective erythropoiesis through intramedullary apoptosis of red cell progenitors, peripheral hemolysis with shortened red cell survival, and severe microcytic hypochromic anemia [1,3,4]. Patients with transfusion-dependent β-thalassemia major typically present in the first year of life with profound anemia, failure to thrive, hepatosplenomegaly, frontal bossing, maxillary hyperplasia, progressive skeletal deformities from marrow expansion, and hypersplenism [1,5]. Without regular transfusions, these patients face life-threatening complications, while lifelong transfusion therapy, although essential for survival, introduces secondary iron overload that progressively damages the myocardium, liver, endocrine organs, and bone [1,6,7,8].

Globally, β-thalassemia is among the most common inherited hemoglobin disorders, particularly in the Mediterranean basin, the Middle East, the Arabian/Persian Gulf, the Indian subcontinent, Southeast Asia, and parts of sub-Saharan Africa. Its wide geographic distribution is largely attributed to historical and endemic factors, with carrier rates in endemic regions commonly ranging from 1% to 5% [9,10,11].

In Iraq, β-thalassemia constitutes a major public health challenge, with a substantial number of registered cases and a particularly high burden in some southern governorates [12,13,14]. Of all hemoglobinopathies, thalassemia accounted for 75% of cases in Iraq [14]. High rates of consanguineous marriage contribute to the persistence and transmission of the disease within families and communities [12,13,15]. The continued occurrence of new cases, together with limitations in prenatal screening and prevention programs, further increases the clinical and economic burden of the disease in the country [12,13].

At the molecular level, β-thalassemia exhibits genetic heterogeneity, with more than 400 HBB variants reported worldwide. Their distribution varies by ethnic background, geographic origin, and population structure, highlighting the diversity of the disease [10,11,16,17,18]. Recent studies have shown substantial regional differences in HBB variant spectra and linked some pathogenic variants to clinical severity, underscoring the importance of local molecular data [19,20,21]. Among the mutations commonly reported in Iraqi and neighboring populations are CD39 (c.118C>T) and IVSI-1 (c.92+1G>A), which are typically associated with β^0^-thalassemia because they create premature termination or abolish normal splicing, respectively, whereas IVSI-6 (c.92+6T>C) is more often associated with β^+^ thalassemia because it causes a partial splicing defect and allows residual β-globin production [17,22]. Intronic and splice-site variants are particularly important because they can significantly alter HBB gene expression, mRNA processing, and phenotypic severity [22,23]. Knowledge of the HBB mutation spectrum is essential for accurate diagnosis, genetic counseling, and the development of targeted therapies, including gene therapy and fetal hemoglobin–inducing agents [24]. Regional variation in the frequency of these mutations highlights the importance of local molecular studies for improving carrier detection, genetic counseling, and prevention strategies [16,23,25].

Repeated blood transfusions, although indispensable for survival in severe β-thalassemia, lead to progressive iron overload and oxidative stress [6,26]. Excess iron promotes the generation of reactive oxygen species, which damage cellular lipids, proteins, and DNA, thereby contributing to tissue injury and long-term complications [6,27]. Antioxidant defense systems therefore play an important role in modulating disease severity [28,29,30]. Catalase is a key antioxidant enzyme that decomposes hydrogen peroxide into water and oxygen, thereby protecting cells against oxidative stress [28,29,30]. The *CAT* polymorphisms rs1001179 C>T and rs7943316 (A>T) are located in regulatory regions and have been reported to influence catalase expression or activity, which may modify oxidative stress susceptibility and disease complications in β-thalassemia [6,30]. Because reactive oxygen species can damage lipids, proteins, DNA, and cellular membranes, impaired CAT activity may also affect the liver, heart, endocrine organs, and spleen. Reports on these variants remain heterogeneous across populations, with some studies showing significant associations and others finding no meaningful relationship, suggesting that their effects may depend on ethnic background, allele frequency, sample size, and co-inherited genetic or environmental modifiers [31,32,33,34,35,36,37].

Accordingly, the present study aimed to characterize the frequency of *HBB* mutations (IVS-I-6, IVS-I-1, and CD39) in 100 transfusion-dependent β-thalassemia patients and 50 controls from Al-Diwaniyah Governorate, Iraq, while analyzing *CAT* gene polymorphisms and oxidative stress markers. The findings identified rs1001179 as a disease-associated oxidative modifier, whereas IVS-I-1 and CD39 (β^0^) mutations were associated with reduced catalase activity despite comparable hematological profiles. These results support the relevance of a precision medicine approach in populations with high consanguinity. By integrating HBB variant analysis with *CAT* polymorphism data, this study provides a dual genetic perspective that may enhance understanding of disease heterogeneity, refine genotype–phenotype correlations, and inform regional screening, genetic counseling, and prevention strategies.

## 2. Materials and Methods

### 2.1. Study Subjects

The study was conducted in strict accordance with the ethical principles of the Declaration of Helsinki and the guidelines governing medical research. Thalassemia cases were investigated, and blood samples were collected from the Thalassemia Center at the Women’s and Children’s Teaching Hospital, Al-Diwaniyah, Iraq, from October 2022 to December 2022. Administrative approval, in the form of a mission facilitation letter, was obtained from the Directorate of the College of Medicine, which facilitated access to the center, patient recruitment, and sample collection from both patients and healthy controls. The study protocol, along with the participant information sheet and consent forms, was reviewed and approved by the Institutional Review Board/Ethics Committee of the College of Medicine, University of Al-Qadisiyah. Informed consent was obtained from all adult participants (patients and healthy controls) before enrollment and before any biological samples were collected. For participants under 18 years of age, informed assent was obtained from the child, and informed consent was secured from their parents or legal guardians.

This study was designed as a retrospective case–control study involving 150 participants in total. These participants were systematically divided into two distinct groups: the Thalassemic Patient Group and the Healthy Control Group.

#### 2.1.1. Thalassemic Patient Group (Case Group)

The case group consisted of 100 patients who were diagnosed with transfusion-dependent β-thalassemia major (TDM). The patients were being routinely managed for the clinical symptoms and manifestations of the disease and were dependent on regular blood transfusions. These patients were recruited from the Thalassemia center in Al-Diwaniyah, Iraq, encompassing individuals of different ages and communities across various regions and villages.

The age of the patients ranged from 1 to 35 years. The gender distribution was recorded as 43 (43.0%) males and 57 (57.0%) females. Prior diagnosis of β-thalassemia major had been established through a combination of complete blood count (CBC), blood film analysis, and hemoglobin electrophoresis.

Clinical data and personal information were also meticulously collected for each patient, including family history, date of birth, living conditions, education, date of first diagnosis, number of blood transfusions received, chelation therapy status, smoking history, date of death (where applicable), and history of splenectomy. Furthermore, relevant ratios and concentrations were measured and compared to normal reference ranges.

#### 2.1.2. Control Group

Controls were recruited from unrelated students at the Al-Diwaniyah Technical Institute (Al-Diwaniyah, Iraq), matched to patients by age, sex, and geographic origin, with no known consanguinity or family relationship to the patient group. Control status was determined based on a prior CBC, and individuals with any abnormal hematological findings, including minor deviations from the reference range, were excluded. However, carrier status was not assessed through molecular screening prior to inclusion.

### 2.2. Exclusion Criteria

For all participants (both patients and controls), those diagnosed with Hepatitis C or Hepatitis B were systematically excluded from the study.

### 2.3. Blood Sample Collection

#### 2.3.1. Samples for Molecular and Hematological Analysis

Venous blood (2 mL) was collected from all participants and stored in EDTA-anticoagulant tubes for molecular analyses and CBC. Samples were stored at −20 °C until processing.

#### 2.3.2. Samples for Biochemical Analysis

Venous blood (3 mL) was collected from study participants into serum gel tubes without an anticoagulant. The tubes were incubated at room temperature for 20 min to allow for clotting, followed by centrifugation at 3000 rpm for 10 min. Supernatant serum was aliquoted into Eppendorf tubes and stored for quantification of ferritin, lipid profile, catalase (CAT), and malondialdehyde (MDA).

### 2.4. Hematological Analysis

Hematological parameters, including Red Blood Cell (RBC) count, White Blood Cell (WBC) count, Hemoglobin (HGB), Mean Corpuscular Volume (MCV), Mean Corpuscular Hemoglobin (MCH), Mean Corpuscular Hemoglobin Concentration (MCHC), Hematocrit (HCT), and platelet count, were determined using the Sysmex XP-300 (Sysmex CorporationTM, Kobe, Japan) hematology analyzer according to the manufacturer’s instructions.

### 2.5. Biochemical Analysis

#### 2.5.1. Serum Ferritin

Serum ferritin concentrations were quantified using the VIDAS automated immunoanalyzer (Biomerieux, Marcy-l’Étoile, France). The enzyme-linked fluorescent assay (ELFA) employed a competitive immunoassay with fluorescence detection at 450 nm (Biomerieux, Marcy-l’Étoile, France). All steps, including reagent dispensing in sealed strips, were performed automatically.

#### 2.5.2. Antioxidant Status

- Catalase activity: CAT activity in serum was measured following [38], based on hydrogen peroxide decomposition. Enzyme efficiency was assessed by the decrease in absorbance at 452 nm.
- Malondialdehyde levels: Serum MDA levels, as a marker of lipid peroxidation, were determined using the thiobarbituric acid reactive substances (TBARS) assay [39]. Samples were measured colorimetrically at 530–540 nm (or fluorometrically at excitation 515 nm/emission 555 nm) under acidic conditions.

#### 2.5.3. Lipid Profile

Serum levels of total cholesterol (TC), triglycerides (TG), High-Density Lipoprotein (HDL), and Low-Density Lipoprotein (LDL) were measured using the DRI-CHEM NX500 Dry chemistry analyzer (Fujifilm, Ratingen, Germany) per manufacturer protocols.

### 2.6. Genomic DNA Extraction and SNP Genotyping

Genomic DNA was extracted from EDTA-blood samples using the gSYAN DNA Extraction Kit (Frozen Blood; Geneaid, New Taipei City, Taiwan ) per manufacturer instructions. Purity was confirmed via NanoDrop (Thermo Fisher, Waltham, MA, USA) at 260/280 nm.

After primer design using WASP (https://bioinfo.biotec.or.th/WASP/home) (accessed on 15 January 2022), the genotyping of *CAT* polymorphisms (rs1001179 C>T and rs7943316 A>T) was performed by Amplification Refractory Mutation System Polymerase Chain Reaction (ARMS-PCR) using the primers listed in Table S1 and PCR conditions optimized with GoTaq G2 Green Master Mix (Promega Corporation, Madison, WI, USA).

The *HBB* mutations CD39 (C>T), IVS1-1 (G<A), and IVS1-6 (T>C) were genotyped by Sequence Specific Primers Polymerase Chain Reaction (SSP-PCR) using primers reported by Gomes et al. 2017 [40] and GoTaq G2 Green Master Mix (Table S2).

### 2.7. Statistical Analysis

Descriptive analyses were performed to assess the general characteristics of the participants (control and patient groups). Numerical variables are described by their mean and standard deviation. Categorical variables are described by their frequencies and compared between groups using the Chi-squared test. The association between sociodemographic and clinical parameters and disease status was determined using univariable logistic regression analysis. Data normality was assessed using the Shapiro–Wilk test. All variables were log-transformed prior to regression analyses.

An analysis of variance (ANOVA) was performed to analyze differences in hematological parameters, CAT levels and MDA levels according to the genotypes of patients with HBB. The effects of HBB genotypes on hematological parameters and the influence of *CAT* polymorphisms on CAT and MDA levels were assessed using multivariate linear regression. Spearman correlations were performed between *CAT* polymorphisms, oxidative stress parameters, and the number of transfusions in patients.

Principal Component Analysis (PCA) was restricted to the three HBB mutations studied in the present study to explore clustering trends based on their reported allele frequencies in different populations. Because the source studies used varying genotyping methods and sampling strategies, this analysis was interpreted as an exploratory visualization of allele frequency similarity rather than formal proof of population genetic structure. Statistical analyses were performed using SPSS v.20 Statistics software (IBM; Armonk, NY, USA). For genetic analysis, allele and genotypic distributions, Hardy–Weinberg Equilibrium (HWE) and association studies were performed using the SNPassoc package in R language (version 4.4.3). For polymorphisms not conforming to HWE, the Cochran–Armitage test was used. A two-sided significance level of *p*-value less than 0.05 was applied.

## 3. Results

### 3.1. General Characteristics

The general characteristics of the 150 participants, including the results of the hematological parameters, lipid and Antioxidant profiles, are summarized in Table 1. The 100 beta-thalassemia patients consisted of 43 males and 57 females, with a mean age at diagnosis of 15.22 ± 6.658 years, resulting in a male/female ratio of 0.75. The control group included 50 participants; 23 of them were women with a mean age of 17.38 ± 6.524 years. No significant differences in smoking status between case and control were found (*p* = 0.449). None of the healthy subjects had any family history of disease; 80% of patients had a family history of beta-thalassemia (*p* < 0.001, OR = 5, CI 95% [3.379; 7.400]). Eighty patients out of 100 were from consanguineous marriages, 49 of which were first-degree.

Seventy-four percent (74/100) of beta-thalassemia patients are found to reside in rural areas, whereas none of the control participants are reported to live in these regions. Beta-thalassemia patients were significantly more likely to reside in rural areas than controls (*p* < 0.001; OR = 3.846, 95% CI: 2.763–5.353). Regarding education level, patients showed a higher proportion of low education (60.6% vs. 30%) and medium education (29.3% vs. 34%), together with a lower proportion of highly educated individuals (10.1% vs. 36%), compared with controls. Accordingly, low education level was more frequently observed among patients than controls (*p* = 0.0044; OR = 2.345, 95% CI: 1.029–5.344). Similarly, poor socioeconomic status was significantly more frequent among patients than among controls (*p* < 0.0001; OR = 15.357, 95% CI: 6.175–38.196). Thirteen patients underwent a splenectomy (Table 1).

Regarding hematological parameters, patients had lower levels of erythrocytes (RBCs), hematocrit (HCT), mean corpuscular hemoglobin (MCH), and mean corpuscular hemoglobin concentration (MCHC) compared to controls (Table 1). Conversely, controls had lower levels of platelets, WBCs, LYMs, NEUTs, and ferritin (*p* < 0.001). Furthermore, patients had lower levels of total cholesterol (TC), HDL, LDL and catalase (CAT) and higher levels of TG and MDA (3.76 ± 1.41 vs. 3.02 ± 0.44) compared to controls (*p* < 0.001). No statistically significant differences were observed for LYM and NEUT between patients and controls (*p* > 0.05) (Table 1).

### 3.2. Genetic Analysis of HBB Gene

#### 3.2.1. Frequencies of HBB Mutations in Controls and Patients

Three prevalent mutations in the *HBB* gene, IVSI-1 (G>A), IVSI-6 (T>C), and CD39 (C>T), were analyzed in 100 patients and 43 controls. It should be noted that genotype data for 7 controls were missing. The frequencies of these mutations are presented in Table 2.

- IVSI-1 (G>A) mutation

The genotype distribution of the IVSI-1 (G>A) mutation differed between controls and patients. In the control group, the G/G genotype was the most frequent (77%), followed by G/A (23%), while no A/A genotype was detected. In patients, the frequency of the G/G genotype decreased to 68%, whereas A/A increased to 26% and G/A accounted for only 6% of cases. In addition, the mutant A allele was more common among patients than controls (29% versus 12%) (Table 2).

- IVSI-6 (T>C) mutation

For the IVSI-6 (T>C) mutation, controls mostly had the T/T genotype (86%), while 14% were T/C, and no C/C genotype was observed. In the patient group, the frequency of T/T was 70%, the C/C genotype was observed in 21% of cases, and T/C in 9%. The C allele was more frequent in patients (25.5%) than in controls (7%) (Table 2).

- CD39 (C>T) mutation:

The CD39 (C>T) mutation also revealed marked differences between the two groups. In controls, the C/C genotype was the most frequent (86%), followed by the C/T genotype (14%), and no T/T genotypes were observed. In patients, the frequency of the C/C genotype was 71%, while that of the T/T genotype reached 22% and that of the C/T genotype 7%. The mutated T allele was much more frequent in patients (25.5%) than in controls (7%) (Table 2).

These three mutations are relatively frequent in the control population, with 23% of individuals carrying the IVSI-1 (G>A) heterozygous genotype, 14% carrying the IVSI-6 (T>C) heterozygous genotype, and 14% carrying the CD39 (C>T) heterozygous genotype. These findings help explain the high frequencies observed in patients, among whom 69 out of 100 carried at least one copy of the three studied mutations.

Among the 43 control individuals, two were found to carry two *HBB* mutations: IVS-I-1 (G>A) with IVS-I-6 (T>C), and IVS-I-1 (G>A) with CD39 (C>T). These individuals are likely compound heterozygotes, as they are clinically asymptomatic. This suggests that the mutations may be located on the same allele (in cis) or that at least one of the variants has a mild or silent effect. However, without haplotype analysis, the phase of these mutations (cis or trans) cannot be definitively determined. No individual was found to carry all three mutations.

#### 3.2.2. Frequencies of HBB Genotype Classes in Patients

Taking into account all three studied mutations, combined genotypes are listed in Table 3. We found 38 patients with simple homozygous β0 or β+ mutations: IVSI-1 (G>A) (β0/β0; n = 17), IVSI-6 (T>C) (β+/β+; n = 10), and CD39 (C>T) (β0/β0; n = 11).

We also observed the concomitant presence of several mutations in some patients. Indeed, 20 patients showed different combined β0/β0, β+/β+, or β+/β0 genotypes, carrying homozygous and/or heterozygous IVS1-1 (G>A), IVS1-6 (T>C), and CD39 (C>T) mutations. We also identified 11 compound heterozygous patients carrying only one of the three studied mutations: IVS1-1 (G>A) (n = 4), IVS1-6 (T>C) (n = 4), and CD39 (C>T) (n = 3). Meanwhile, no mutation was identified in 31 patients. Based on their typical transfusion-dependent β-thalassemia phenotype, we expect that these patients carry other HBB mutations in different β0/β0, β+/β+, and/or β+/β0 genotypes (Table 3).

#### 3.2.3. Analysis of HBB Genotypes and Hematological Data in Patients

The hematological parameters of each genotype group (simple homozygous mutations, combined *HBB* mutations, compound heterozygous mutations, and uncharacterized mutations) in the 100 patients are presented in Table 4. No significant differences in hematological parameters, CAT, or MDA levels were observed among the different genotype groups (ANOVA, *p* > 0.05).

Similarly, RBC count, MCV, and MDA values did not differ significantly among the groups. Although compound heterozygous patients tended to show the lowest RBC, HGB, HCT, MCV, MCH, and platelet values, these differences did not reach statistical significance. In contrast, patients with simple homozygous mutations showed the lowest values of WB10, NEUT, ferritin, CAT, MDA, and number of transfusions. Likewise, although patients with combined *HBB* mutations tended to have lower LYM levels, no statistically significant difference was observed compared with the other groups (Table 4).

A multiple linear regression model was used to assess the association between HBB mutation groups and hematological parameters, CAT, and MDA levels, adjusting for age and gender. The analysis included the 100 patients and considered β+ (IVS1-6 homozygous) (n = 10) and β0 (IVS-I-1 or CD39 homozygous) genotypes as the predictors of interest. The analysis reveals that the β0 mutation group) (n = 28), specifically the co-occurrence of IVS-I-1 (G>A) and CD39 (C>T), was associated with lower CAT levels (standardized β = −0.205; *p* = 0.046) (Table S3). Age also showed a negative association with CAT levels (standardized β = −0.250; *p* = 0.013), suggesting that increasing age was associated with a moderate decline in catalase activity after adjustment for other covariates.

#### 3.2.4. Distribution of Different Frequencies of HBB Mutations (IVSI-1 (G>A) IVS1-6 (T>C) and CD39 (C>T)) in the Iraq Region and Neighboring Areas

Table S4 summarizes the frequencies of three β-globin gene mutations, IVSI-1 (G>A), IVSI-6 (T>C), and CD39 (C>T), across Iraqi Arab and Kurdish patients with thalassemia from various studies and data from neighboring Middle Eastern countries, worldwide regions, and the global HbVar database. In our cohort, the allele frequencies of IVS-I-1 (G>A), IVS-I-6 (T>C), and CD39 (C>T) were 29%, 25.5%, and 25.5%, respectively. These values are higher than those reported in several other Iraqi populations, including Karbala, Baghdad, and Iraqi Kurdish regions, where different mutations may predominate. Internationally, comparable or higher frequencies of individual variants have been reported in some countries, such as Tunisia, Bahrain, and Lebanon, but the relatively balanced and simultaneously high frequencies of all three mutations observed in Al-Diwaniyah appear less common. This pattern may reflect local founder effects and consanguinity and supports the value of a regionally tailored screening panel.

These frequencies were used to generate a PCA that clusters populations based on similarities in mutation rate frequencies. The PCA coordinates (PC1: 46% variance, PC2: 33%) are derived from the allele frequency reported in Table S4, where populations are positioned according to their IVS1-6 (T>C), IVS1-1 (G>A), and CD39 (C>T) mutation profiles.

**Figure 1:**
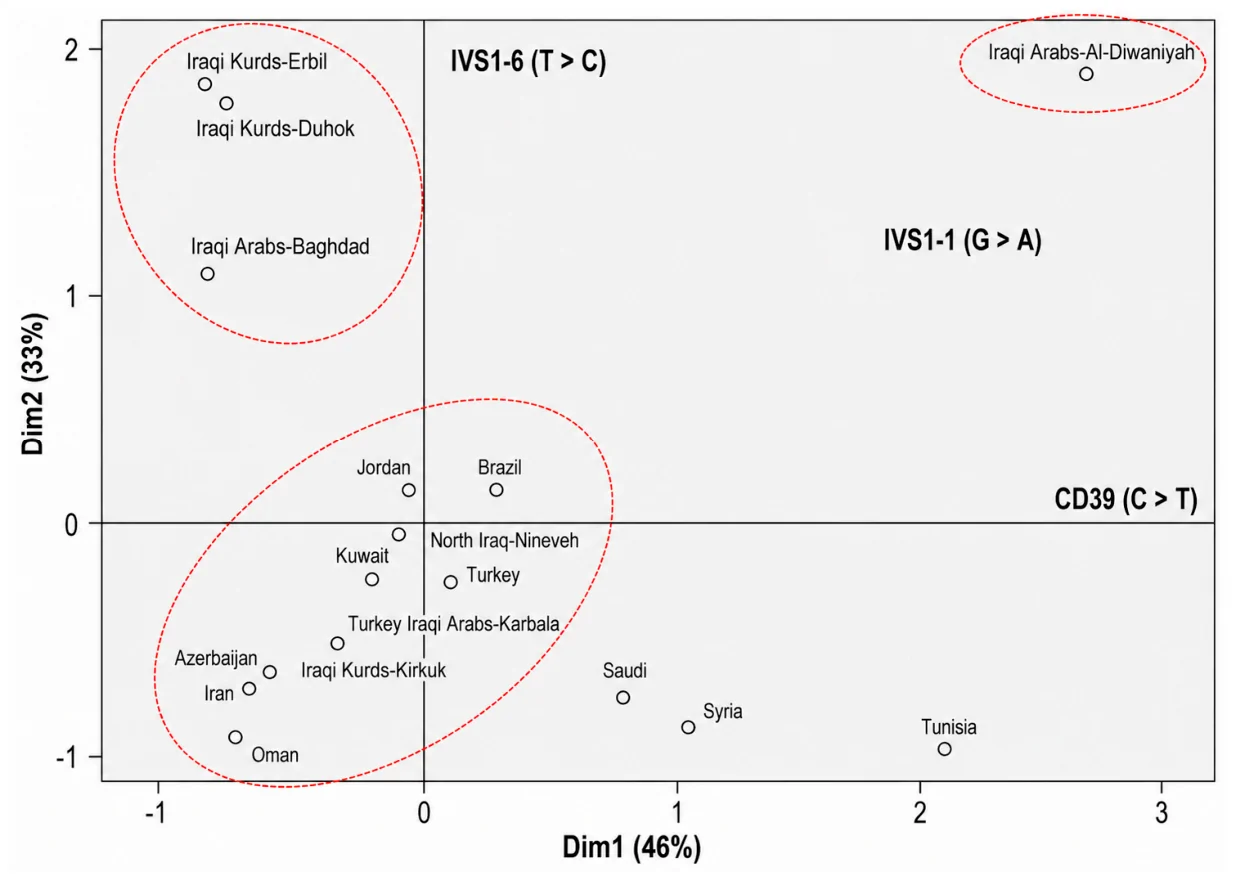
Principal Component Analysis of β-Thalassemia Mutation Frequencies (IVSI-1 (G>A), IVSI-6 T>C), CD39 (C>T)) across Middle Eastern Populations. Red dashed ellipses are shown for visual guidance only to highlight populations with similar mutation distribution patterns; they do not represent statistically defined clusters.

The PCA biplot provides an exploratory visualization of allele frequency similarities between different populations reported for our β-thalassemia cohort (Figure 1), based on the relative frequencies of three common HBB variants. The plot suggests that Iraqi Kurds (Erbil/Dohuk) and Iraqi Arabs (Al-Diwaniyah) show broadly similar allele frequency profiles, positioned together in the upper right region, largely driven by relatively high IVS-I-6 (T>C) frequencies, while Iraqi Arabs from Baghdad occupy an intermediate position. Our study (200 chromosomes) reports notably high rates (25.5–29%) in Al-Diwaniyah Iraqi Arabs versus other studies. Iraqi Arabs from Al-Diwaniyah exhibit the highest prevalence of the IVS1-6 (T>C) (25.5%), consistent with the finding of this study. Iraqi Kurds from Erbil/Duhok show rates of 33.1% to 32.4%, respectively. Other Iraqi groups have rates ranging from 2% to 9.6%. A large West Asian group (Azerbaijan/Iran/Oman/Turkey) occupies the lower left portion of the graph, with transitional populations (Jordan/Brazil/North Iraq/Kuwait) in the center. In neighboring countries such as Syria, the frequency of the IVS1-1 (G>A) mutation reaches 9.2%. In contrast, in Mediterranean countries like Tunisia, the frequency of the CD39 (C>T) mutation is 48.76%, which distinguishes Tunisia from other countries on the PCA graph (Figure 1).

#### 3.2.5. Other Studied HBB Mutations in the Iraq Region and Neighboring Areas

**Figure 2:**
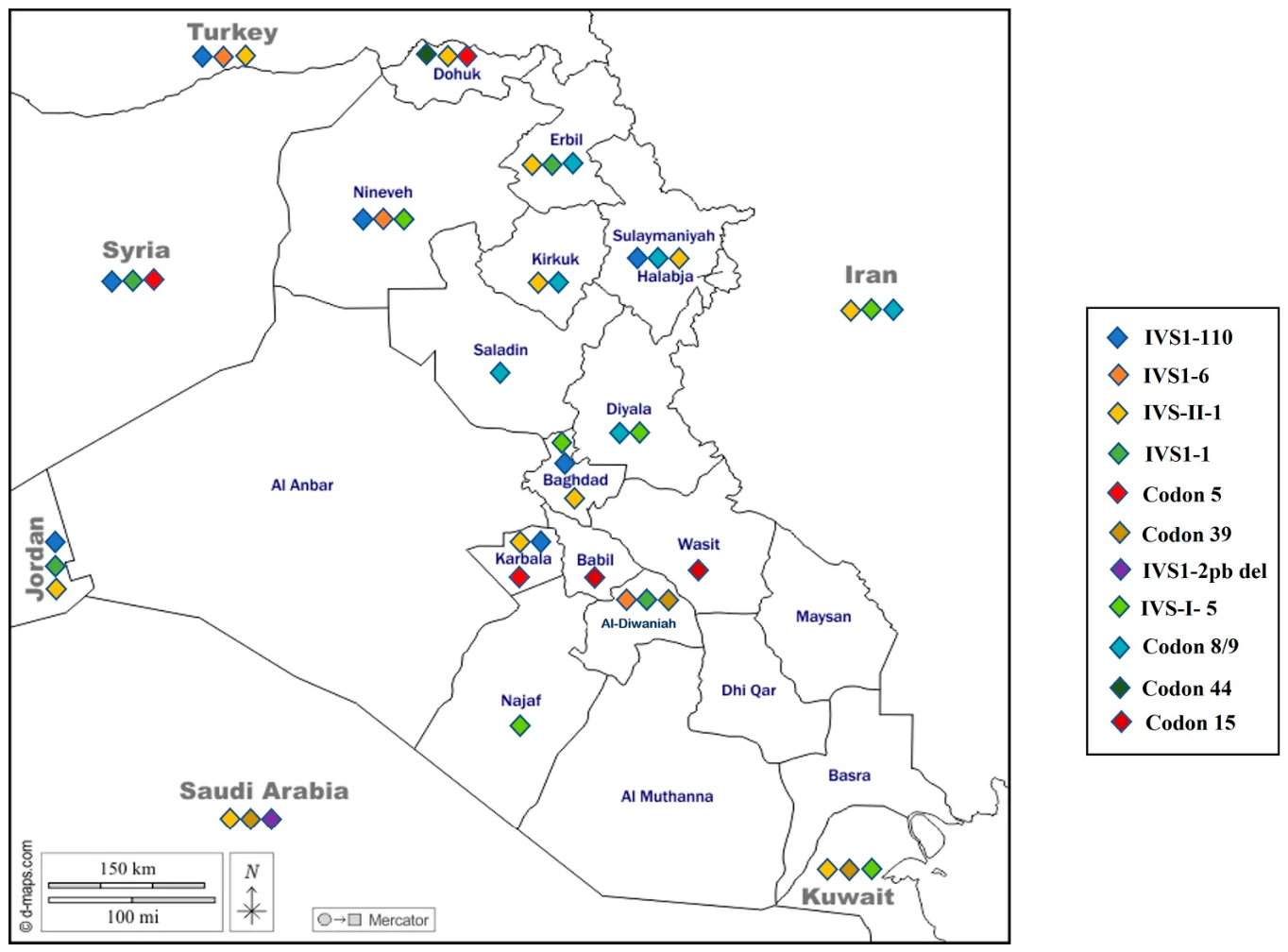
A map of Iraq showing the most frequent mutations in Al-Diwaniah province and other provinces in Northern Iraq, as well as surrounding countries. Base map adapted from D-Maps with modifications (original map available at: https://www.d-maps.com/carte.php?num_car=4295&lang=fr; accessed on 2 June 2026).

In addition to the mutations analyzed (IVSI-1 (G>A), IVSI-6 T>C), CD39 (C>T), other HBB variants have been identified in thalassemia patients across various populations in other studies. Figure 2 illustrates the geographical distribution of *HBB* gene mutations (IVSI-110, IVSI-6, IVSII-1, IVSI-1, codon 5, codon 39, IVSI-2pb del, IVSI-5, codon 8/9, codon 44, and codon 15) across Iraq and its neighboring regions, including Turkey, Syria, Iran, Jordan, Saudi Arabia, and Kuwait. Different colored symbols represent distinct *HBB* mutations, showing their presence and variation across multiple Iraqi provinces such as Baghdad, Nineveh, Basra, and Erbil. The most prevalent mutations among those studied were IVS2-1 and IVS1-110.

### 3.3. Genetic Analysis of CAT Genes

#### 3.3.1. Association Between Polymorphism in Catalase Gene (rs1001179 C>T and rs7943316 A>T) with Beta-Thalassaemia

Catalase gene is a key antioxidant enzyme that breaks down hydrogen peroxide; polymorphisms in *CAT* can alter enzyme activity or expression, thereby affecting the capacity to handle oxidative stress and may act as a modifier of the severity or phenotype in HBB related hemoglobinopathies. Two prevalent polymorphisms in the *CAT* gene: rs1001179 C>T and rs7943316 A>T were analyzed in 100 patients and 50 controls. These two SNPs were significantly deviated from HWE (*p* = 0.032 and *p* = 0.042 for rs1001179 C>T and rs7943316 A>T, respectively).

The distribution of genotypes and alleles of these two SNPs among patients and controls is presented for the codominant, dominant and recessive models, respectively, in Table 5.

A significant difference in genotype distribution for rs1001179 C>T was observed between cases and controls under the dominant and codominant models. The (C/T) genotype is significantly more frequent in patients (33%) than in controls (14%), with an OR of 3.25 (95% CI: 1.31–8.07), indicating a higher risk of beta-thalassemia for heterozygotes. A significant association between patients carrying at least one T allele (C/T+T/T vs. C/C) was found with an OR of 2.90 (95% CI: 1.30–6.44), *p* = 0.006139. A significant allelic association between the T allele and patient status was found (*p* = 0.016).

For rs7943316 A>T, no difference in the allele and genotype frequency between cases and controls was found in any genetic model (all *p*-values are > 0.05) (Table 5).

#### 3.3.2. Correlation Analysis of CAT Polymorphisms, Oxidative Stress Parameters and Number of Transfusions in Patients

Only the correlation between *CAT* and the number of transfusions is statistically significant and negative (r = −0.203, *p* = 0.043). This suggests a weak but real association, where an increase in transfusions is linked to a decrease in CAT rate. There was no association between *CAT* gene polymorphisms and concentrations (MDA and CAT). In fact, age has a negative and significant effect on CAT concentration; as age increases, CAT decreases slightly (*p* = 0.018) (Table S4).

## 4. Discussion

β-thalassemia is an inherited hemoglobin disorder caused by mutations in the *HBB* gene that reduce or abolish β-globin chain synthesis, leading to ineffective erythropoiesis, chronic anemia, and variable clinical severity [41]. The present study included 100 transfusion-dependent β-thalassemia patients and 50 healthy controls from South Iraq, allowing comparison of clinical, hematological, and biochemical features between affected and non-affected subjects.

Our results showed that β-thalassemia was strongly clustered in socially and genetically vulnerable groups: 61.25% of patients had a first-degree family history of the disease, 74% were from rural areas, 60.6% had low educational attainment, 71.4% belonged to a poor socioeconomic background, and 80% were born from consanguineous marriages. This pattern indicates that the burden of β-thalassemia in South Iraq is reinforced by the high frequency of disease transmission within closely related families. Given the monogenic nature of the disorder, such socioeconomic and demographic variables should not be interpreted as direct causal risk factors, but rather as markers of population structure reflecting persistent consanguineous unions that favor transmission of deleterious HBB alleles [42,43]. This interpretation is consistent with previous reports identifying β-thalassemia as a major public health concern in Iraq and neighboring countries, particularly in settings where consanguinity remains common [15,42,43,44]. Beyond disease occurrence, these same factors may indirectly influence disease progression and outcomes. Transfusion-dependent patients require regular access to healthcare services, including monitoring, transfusion support, and iron chelation therapy. In rural or socioeconomically disadvantaged settings, limited access may delay diagnosis, reduce carrier screening, and weaken preventive efforts such as premarital counseling [41,45]. Although these barriers do not alter the underlying genetic basis of β-thalassemia, they may contribute to complications such as iron overload, splenic pathology, cardiac dysfunction, and endocrine failure. Overall, the high frequency of familial cases likely reflects both autosomal recessive inheritance and marriage/residence patterns that shape allele transmission and access to care.

Hematologically, patients with β-thalassemia had significantly lower RBC, HGB, HCT, MCV, MCH, and MCHC values than controls, a profile that is typical of ineffective erythropoiesis, microcytosis, and hypochromia. These abnormalities arise because defective β-globin synthesis leads to excess unmatched α-globin chains, which damage developing erythroid cells, impair erythropoiesis, and shorten red cell survival [5,46,47]. In contrast, the elevated platelet and white blood cell counts observed in our cohort likely reflect chronic marrow stress and compensatory hematopoietic activation [47,48,49,50]. Recurrent transfusion exposure, chronic inflammation, and cytokine-mediated marrow stimulation may also contribute to leukocytosis in these patients [49,51,52]. In addition, markedly elevated ferritin levels were consistent with repeated blood transfusions and ongoing hemolysis, two features typical of severe transfusion-dependent β-thalassemia [53,54,55,56]. We did not assess liver function (AST, ALT), glycemic status (fasting glucose, HbA1c), or renal function (BUN, creatinine), as these parameters were outside the primary scope of the present investigation, which focused on the HBB mutation spectrum and CAT gene polymorphisms in relation to oxidative stress. We acknowledge that these clinical parameters are relevant to the long-term management of transfusion-dependent β-thalassemia patients, given the known risk of iron-overload-related hepatic, endocrine, and renal complications, and we recommend that future studies incorporate these markers to provide a more comprehensive clinical characterization of the cohort.

Our lipid findings showed that β-thalassemia patients had significantly lower total cholesterol, HDL, and LDL levels, together with higher triglyceride concentrations than controls. This dyslipidemic pattern may reflect chronic hemolysis, ineffective erythropoiesis, iron overload, hepatic involvement, and repeated transfusion-related metabolic disturbance, all of which can interfere with normal lipid synthesis, transport, and clearance [47,55,57,58,59,60]. The reduction in HDL and LDL may also be related to increased oxidative stress and altered membrane lipid turnover, whereas elevated triglycerides may indicate impaired lipid metabolism and liver dysfunction [57,58,59,60]. This interpretation is supported by previous studies from Iraq and other populations reporting a similar low-cholesterol, high-triglyceride profile in β-thalassemia major [61,62,63,64,65]. These abnormalities are clinically relevant because disturbed lipid metabolism and oxidative stress are closely interconnected in β-thalassemia and may together contribute to vascular and organ complications.

At the molecular level, our analysis identified IVSI-1 (G>A), IVSI-6 (T>C), and CD39 (C>T) as the principal HBB variants in our cohort of 100 β-thalassemia patients from South Iraq (Al-Diwaniyah), occurring significantly more frequently in patients than in the 43 controls analyzed. The principal HBB variants identified in this study—IVS-I-1 (G>A), IVS-I-6 (T>C), and CD39 (C>T)—are not unique to Al-Diwaniyah but are among the most common β-thalassemia mutations reported across the Mediterranean region, the Middle East, and South Asia [19,21,66,67,68,69,70]. Their frequencies vary among populations, likely reflecting founder effects, migration, and consanguinity. In our cohort, what stands out is the relatively balanced contribution of these variants and the high rate of compound heterozygous and combined genotypes, suggesting a distinct regional mutation pattern. Notably, 69 patients carried at least one copy of these variants, including 38 simple homozygotes, 20 patients with combined genotypes, and 11 compound heterozygotes, while 31 patients (31%) showed no detected variants despite their transfusion-dependent phenotype. Among the identified alleles, IVS-I-1 (G>A), IVS-I-6 (T>C), and CD39 (C>T) accounted for approximately 29%, 25.5%, and 25.5% of mutant chromosomes, respectively, underscoring their major role in the molecular pathogenesis of β-thalassemia in South Iraq. This profile is consistent with previous reports identifying these variants as key β-thalassemia mutations worldwide [17,18]. This supports the use of a targeted screening panel tailored to South Iraq to improve carrier detection, prenatal diagnosis, and clinical management [19,21]. At the same time, the absence of a detectable mutation in nearly one-third of transfusion-dependent patients highlights substantial genetic heterogeneity and represents an important limitation of the present study. Rather than indicating the absence of an underlying molecular defect, this result most likely reflects the restricted targeted HBB panel used here, which included only three mutations selected based on their high frequency reported in the regional literature. Similar studies from neighboring populations have likewise reported a considerable proportion of uncharacterized alleles when testing was limited to a small number of common variants, suggesting that important regional mutations may remain undetected under such an approach [71,72]. In this context, variants such as IVS-I-110, IVS-II-1, and codon 15 (TGG > TGA), which have been described in surrounding cohorts but were not examined in this population, may account for some of the unresolved cases [72,73]. Because broader screening or full HBB sequencing was not performed, the mutation spectrum reported here likely underestimates the true genetic heterogeneity of β-thalassemia in this population.

Among the 43 controls, two carried two HBB mutations and remained asymptomatic, which is consistent with evidence that phenotypically healthy individuals may still harbor HBB variants, including pathogenic or deleterious ones [74]. This suggests that these variants may have limited clinical impact in carriers, but, without haplotype analysis, their phase cannot be determined. Controls were matched to patients by age, sex, and geographic origin, and were selected on the basis of a normal complete blood count (CBC) profile to minimize obvious hematological differences between groups. Given the high prevalence of β-thalassemia in Iraq, estimated at 37.1 per 100,000 population [13], it is plausible that some controls represented unrecognized carriers rather than a fully genetically “neutral” reference population, particularly because molecular screening was not performed at the time of recruitment. This may help explain the relatively high frequencies of IVS-I-1 (G>A), IVS-I-6 (T>C), and CD39 (C>T) mutations observed in the control group, as well as the presence of two controls carrying more than one mutation. Nevertheless, because carrier status is usually clinically silent, this limitation is unlikely to have introduced major systematic bias into the observed odds ratios or the genotype–phenotype associations reported in the study. Such findings reinforce the need for haplotype-based studies to better define mutation inheritance patterns and their relationship to clinical expression [75].

The regional mutation map was included to provide contextual background rather than to specifically illustrate the distribution of the three mutations analyzed in our own cohort, which fell outside the primary scope of the present study in the Iraqi population. Rather, it was designed to provide a broader contextual perspective on HBB gene mutations reported across the region, including the most extensively studied variants, in order to better situate our findings within the wider mutational landscape of this gene. Given the uneven and incomplete sampling across the populations included in the map, the observed patterns should be interpreted with caution and should not be taken as definitive evidence of the true regional distribution of these mutations.

A detailed geographical mutation map likewise revealed pronounced regional differences, showing our study mutations alongside other prevalent HBB variants, especially IVSII-1 and IVSI-110, distributed across Iraqi governorates such as Baghdad, Nineveh, Basra, and Erbil, as well as neighboring countries including Turkey, Syria, Iran, Jordan, Saudi Arabia, and Kuwait. These patterns demonstrate that HBB variant frequencies vary markedly because of founder effects, population stratification, migration, and consanguinity [19,20,21]. Such pronounced heterogeneity limits extrapolating mutation spectra across governorates or borders, emphasizing locally derived data for precise diagnostic and preventive strategies [12,13,16,23,25].

In parallel with the primary HBB analysis, the genetic investigation was extended to oxidative pathways by examining two *CAT* promoter variants as potential disease modifiers. Both CAT polymorphisms (rs1001179 C>T and rs7943316 A>T) showed deviation from Hardy–Weinberg equilibrium in the control group (*p* = 0.032 and *p* = 0.042, respectively). Several factors may explain this finding. First, the modest number of control subjects (n = 50) relative to cases (n = 100) limited the precision of allele-frequency estimates, and small samples are more susceptible to random variation and apparent deviation from HWE, even in the absence of true biological effects. Second, recruiting a closely matched control group was challenging because of the wide age range of the patients and the need to identify individuals from the same geographic background with a normal CBC profile, which may have introduced sampling heterogeneity. Population stratification and non-random mating patterns, both plausible in a population with documented high consanguinity, may also contribute to HWE deviation independent of genotyping quality. Although each genotype call was independently reviewed by two observers, with discrepancies resolved by reexamination before data analysis, formal genotyping quality control procedures—including systematic re-genotyping of a subset of samples to assess reproducibility and the routine use of positive and negative controls—were not implemented in the present study. This limits the ability to fully exclude genotyping error as a contributor to the observed HWE deviation. Given the borderline HWE *p* values, the limited sample size, and the absence of formal genotyping QC metrics, these findings should be regarded as preliminary and confirmed in larger, independently recruited cohorts with well-matched control groups and standardized genotyping quality control procedures, including call rate reporting and blinded re-genotyping.

Genotyping of rs1001179 (C>T) and rs7943316 (A>T) in 100 β-thalassemia patients and 50 controls demonstrated a significant association between rs1001179 (C>T) and disease status, but no association for rs7943316 (A>T). For rs1001179 (C>T), the CT genotype was more frequent among patients than controls (33% vs. 14%; OR = 3.25, 95% CI 1.31–8.07, *p* = 0.019 under a codominant model), and the dominant model also showed increased risk for CT+TT versus CC (OR = 2.90, 95% CI 1.30–6.44, *p* = 0.006). The T allele frequency was higher in patients than controls (25.5% vs. 13%, *p* = 0.016), supporting its role as a risk-associated allele in this cohort. The T allele of rs1001179 (C>T) has been consistently implicated in altered *CAT* gene expression across diverse oxidative stress-related conditions and disease contexts, including hematological malignancies, diabetes-related complications, and chronic inflammatory disorders. This functional promoter variant disrupts normal transcription factor binding, particularly affecting PAX-6 and STAT4 response elements, leading to reduced basal *CAT* transcription rates and consequently diminished catalase enzyme activity. These molecular effects compromise the detoxification capacity of hydrogen peroxide and potentiate oxidative damage through the accumulation of reactive oxygen species. In β-thalassemia major patients, where chronic hemolysis, iron overload, and frequent transfusions already reflect substantial organ burden, this T allele-mediated antioxidant impairment may aggravate disease susceptibility and contribute to complications such as progressive spleen pathology, cardiac dysfunction, and endocrine failure in transfusion-dependent patients.

By contrast, rs7943316 (A>T) showed no significant association with disease susceptibility in the present cohort, in agreement with the recent Iraqi study by Inteek and Madhkhoor (2026) [34], and with findings from Indian and other cohorts reporting no statistically significant association between this polymorphism and β-thalassemia risk [34,35]. Although rs7943316 (A>T) is also located in the *CAT* promoter and may theoretically affect gene expression, published findings remain notably inconsistent across populations. This heterogeneity likely arises from ethnic background differences, variable minor allele frequencies due to regional genetic structure, limited sample sizes reducing statistical power, and disease heterogeneity influenced by co-inherited modifiers and transfusion regimens [32]. Such discrepancies underscore the necessity for larger, multi-ethnic meta-analyses to clarify the true contribution of rs7943316 (A>T) to β-thalassemia oxidative pathology.

Neither SNP showed a direct correlation with measured CAT or MDA levels or with transfusion burden (β ≈ 0, *p* > 0.80), even though β-thalassemia patients overall had significantly lower CAT activity and higher MDA levels than controls. This pattern supports the view that oxidative stress in β-thalassemia is multifactorial and not determined by a single modifier alone. Iron overload, chronic hemolysis, and exposure to repeated transfusions promote the production of reactive oxygen species and lipid peroxidation, which can deplete antioxidant defenses even in the absence of a direct correlation between SNP and biomarker [76,77,78].

This dual HBB–CAT analysis addresses a critical genotype–phenotype gap that underscores our study’s innovation. Despite no significant differences in hematological parameters across HBB genotype groups, simple homozygotes (n = 38), combined mutations (n = 20), compound heterozygotes (n = 11), and uncharacterized cases (n = 31; ANOVA *p* > 0.05), multivariate analysis indicated that IVSI-1 (G>A)/CD39 (C>T) β^0^ mutations were associated with lower CAT levels (β = −0.205, *p* = 0.046), suggesting that these genotypes may exacerbate oxidative stress. This uniform clinical phenotype is consistent with the multifactorial nature of β-thalassemia severity, where modifiers such as increased HbF production and co-inherited α-thalassemia, together with non-genetic factors such as transfusion regimens, often exert stronger influence than the primary HBB genotype alone [17]. Nonetheless, our novel finding that IVSI-1 (G>A)/CD39 (C>T) β^0^ mutations specifically predict reduced CAT activity reveals that distinct HBB lesions can selectively impair antioxidant defenses even when hematological features remain broadly similar. Similar integrated dual-genotyping approaches remain scarce in the region, where Iraqi and Middle Eastern studies have mainly reported either HBB mutation spectra, such as the high IVSI-6 (T>C) frequency in Iraqi Kurds, or isolated *CAT* polymorphisms, but not both together in a combined oxidative framework. Taken together, our results show for the first time that IVSI-1 (G>A)/CD39 (C>T) β^0^ mutations are linked to lower CAT activity, connecting specific HBB genotypes to antioxidant impairment and providing a more integrated view of disease heterogeneity by linking the structural hemoglobin defect to oxidative imbalance.

In this regional context of high consanguinity, the T allele of rs1001179 (C>T) emerges as a clinically relevant oxidative modifier superimposed on primary HBB lesions. Both SNPs deviated from Hardy–Weinberg equilibrium in controls (rs1001179C>T; *p* = 0.032; rs7943316 A>T; *p* = 0.042), likely reflecting low minor allele frequencies and population structure. Taken together, these findings support targeted HBB–CAT genotyping for Middle Eastern cohorts and warrant meta-analyses to resolve the inconsistent role of rs7943316 (A>T), establishing a useful framework for precision β-thalassemia management.

In summary, β-thalassemia in southern Iraq has a considerable clinical and social impact, characterized by hematological abnormalities, lipid disorders, prevalent regional HBB mutations, iron overload, and oxidative stress. These findings align with patterns from Al-Diwaniyah and neighboring cohorts, underscoring the need for targeted molecular screening, genetic counseling, and prevention programs in high-consanguinity populations. Our dual HBB–CAT analysis further highlights opportunities for expanded mutation panels and modifier gene studies to refine precision management strategies.

Although the study population was recruited from a single center in Al-Diwaniyah, the observed findings were consistent with those reported in other Iraqi populations, supporting the robustness of the identified associations. Moreover, this study is among the first to investigate the relationship between catalase gene variants and a severe transfusion-dependent β-thalassemia phenotype, providing novel insights into genotype–phenotype interactions. The sample size was adequate for the primary analyses; however, larger multicenter cohorts would allow for more detailed subgroup investigations and enhance statistical power. Future studies incorporating more comprehensive screening for *HBB* and *CAT* genetic variations could further refine and validate the associations observed in the present work.

## 5. Conclusions

β-thalassemia in South Iraq is characterized by a combination of adverse social determinants, severe hematological and biochemical disturbances, and a distinct regional mutation spectrum. The predominance of IVSI-1 (G>A), IVSI-6 (T>C), and CD39 (C>T) confirms the importance of local HBB screening, while the association of rs1001179 (C>T) with disease and the link between IVSI-1 (G>A)/CD39 (C>T) β^0^ genotypes and reduced catalase activity highlight the role of oxidative stress modifiers. These results underscore the importance of integrating *HBB* and *CAT* analysis to better understand disease heterogeneity and support precision prevention and management.

## Figures and Tables

**Table 1:** Characteristics of Iraqi participants.

**a: Descriptive and Univariate Regression of Qualitative Characteristics**						
**Variables**	**Subgroups**	**Total (%)**	**Controls (n = 50) %**	**Patients (n = 100) %**	***p*** **Value** **(Fisher’s Exact Test)**	**OR [CI 95%]**
Sex	Male	70 (46.7)	27 (54)	43 (43)	0.203	0.643 [0.325; 1.272]
	Female	80 (53.3)	23 (46)	57 (57)		
Familial history	No	70 (46.7)	50 (100)	20 (20)	0.000 ***	**5** [3.379; 7.400]
	yes	80 (53.3)	0 (0)	80 (80)		
Living	Rural	74 (49.3)	0 (0)	74 (74)	0.000 ***	
	Urban	76 (50.7)	50 (100)	26 (26)		3.846 [2.763; 5.353]
Education	low	75 (50.3)	15 (30)	60 (60.6)	0.000 ***	0.326 [0.123; 0.866]
	medium	46 (30.9)	17 (34)	29 (29.3)	0.004 **	2.345 [1.029; 5.344]
	High	28 (18.8)	18 (36)	10 (10.1)		
Socioeconomic status	Poor	77 (52.0)	7 (14)	70 (71.4)	0.000 ***	15.357 [6.175; 38.196]
	rich	71 (48)	43 (86)	28 (28.6)		
Smoking	No	139 (92.7)	48 (96)	91 (91)		
	Yes	9 (6)	2 (4)	7 (7)	0.449	0.542 [0.108; 2.710]
**b: Descriptive and Univariate Regression of Quantitative Characteristics**						
	**Variables**	**Total (%)**	**Controls** **(n = 50)**	**Patients (n = 100)**	***p* Value** **(Student Test)**	**OR [CI 95%]**
	Age	15.94 ± 6.670	17.38 ± 6.524	15.22 ± 6.658	0.061	0.953 [0.905; 1.003]
Blood parameters	RBC	3.659 ± 0.91	4.70 ± 0.68	3.13 ± 0.44	0.000 ***	0.027 [0.08; 0.088]
	HGB	9.614 ± 2.642	13.13 ± 0.891	7.855 ± 0.861	0.000 ***	NS
	HCT	25.85 ± 4.97	30.39 ± 5.45	23.57 ± 2.61	0.000 ***	0.676 [0.598; 0.764]
	MCV	78.65 ± 6.54	83.82 ± 4.90	75.582 ± 5.45	0.000 ***	0.612 [0.510; 0.735]
	MCH	26.04 ± 2.90	30.39 ± 3.61	23.57 ± 2.08	0.000 ***	0.709 [0.604; 0.832]
	MCHC	34.7 ± 3.52	27.59 ± 4.42	25.26 ± 1.53	0.000 ***	0.571 [0.468; 0.697]
	platelet count	400.6 ± 488.37	217.28 ± 55.38	492.21 ± 576.20	0.000 ***	1.015 [1.009; 1.020]
	WBC.10	7.677 ± 3.86	5.67 ± 1.04	8.67 ± 4.34	0.000 ***	1.714 [1.340; 2.194]
	LYM	43.81 ± 12.79	43.858 ± 12.51	43.783 ± 12.99	0.973	1 [0.973; 1.026]
	LYM.10	4.619 ± 8.22	2.30 ± 0.65	5.77 ± 9.88	0.000 ***	1.497 [1.069; 2.096]
	NEUT.	48.08 ± 10.28	44.87 ± 6.63	48.68 ± 11.66	0.228	1.017 [0.984; 1.051]
	NEUT.10	3.391 ± 1.99	2.40 ± 0.80	3.88 ± 2.21	0.000 ***	2.216 [1.513; 3.244]
	Ferritine	1949 ± 2463.685	67.36 ± 41.47	2890 ± 2540.09	0.000 ***	1.536 NS
Lipid profiles	TC	100.81 ± 30.63	129.88 ± 22.64	86.28 ± 22.77	0.000 ***	0.911 [0.882; 0.940]
	TG	97.46 ± 43.16	77.04 ± 37.31	107.67 ± 42.42	0.000 ***	1.020 [1.010; 1.031]
	HDL	30.11 ± 14.91	44.76 ± 14.73	22.79 ± 8.05	0.000 ***	0.840 [0.792; 0.890]
	LDL	48.69 ± 21.40	68.48 ± 20.66	38.80 ± 13.47	0.000 ***	0.906 [0.878; 0.934]
Antioxidant profiles	CAT	0.914 ± 0.40	1.16 ± 0.39	0.79 ± 0.34	0.000 ***	0.064 [0.21; 0.198]
	MDA	3.517 ± 1.23	3.02 ± 0.44	3.76 ± 1.41	0.000 ***	2.466 [1.390; 4.374]

None of the patients or controls had a history of alcohol consumption. n: number ***: *p* < 0.0001; **: *p* < 0.001; RBC: Red Blood Cell, HGB: Hemoglobin, HCT: Hematocrit, MCV: Mean Corpuscular Volume; MCH: Mean Corpuscular Hemoglobin, MCHC: Mean Corpuscular Hemoglobin Concentration, WBC.10: White Blood Cells (×10^3^/μL), LYM: Lymphocytes, LYM.10: Lymphocytes (×10^3^/μL), NEUT: Neutrophils, NEUT.10: Neutrophils (×10^3^/μL), TC: Total Cholesterol, TG: Triglycerides, HDL: High-Density Lipoprotein, LDL: Low-Density Lipoprotein, CAT: Catalase, MDA: Malondialdehyde level.

**Table 2:** Frequencies of the IVSI-1 (G>A), IVSI-6 (T>C) and CD39 (C>T) mutations in the HBB gene among patients and controls.

Mutation		Controls (n = 43) n (%)	Patients (n = 100) n (%)
IVSI-1 (G>A)			
Genotypes	G/G	33 (77)	68 (68)
	G/A	10 (23)	6 (06)
	A/A	0	26 (26)
Alleles	G	76 (88)	142 (71)
	A	10 (12)	58 (29)
IVSI-6 (T>C)			
Genotypes	T/T	37 (86)	70 (70)
	T/C	6 (14)	9 (09)
	C/C	0	21 (21)
Alleles	T	80 (93)	149 (74.5)
	C	6 (7)	51 (25.5)
CD39 (C>T)			
Genotypes	C/C	37 (86)	71 (71)
	C/T	6 (14)	7 (07)
	T/T	0	22 (22)
Alleles	C	80 (93)	149 (74.5)
	T	6 (7)	51 (25.5)

**Table 3:** Frequencies of β-Globin genotypes IVS1-1 (G>A), IVS1-6 (T>C), and Codon 39 (C>T) in 100 TDM Patients.

Mutations	Genotype	Type	Number
Simple homozygous mutations	IVS1-1 (G>A)/IVS1-1 (G>A)	β^0^/β^0^	17
	IVS1-6 (T>C)/IVS1-6 (T>C)	β^+^/β^+^	10
	CD39 (C>T)/CD39 (C>T)	β^0^/β^0^	11
**Sub-total**			**38**
Combined HBB mutations	IVS1-1 (G>A)/IVS1-1 (G>A) + IVS1-6 (T>C)/IVS1-6 (T>C)	β^0^/β^0^	2
	IVS1-1 (G>A)/IVS1-1 (G>A) + IVS1-6 (T>C)/IVS1-6 (T>C) + CD39 (C>T)	β^0^/β^0^	1
	IVS1-1 (G>A)/IVS1-1 (G>A) + CD39 (C>T)/CD39 (C>T)	β^0^/β^0^	3
	IVS1-1 (G>A)/IVS1-1 (G>A) + IVS1-6 (T>C)	β^0^/β^0^	2
	IVS1-1 (G>A)/IVS1-1 (G>A) + CD39 (C>T)	β^0^/β^0^	1
	IVS1-1 (G>A) + IVS1-6 (T>C)/IVS1-6 (T>C) + CD39 (C>T)/CD39 (C>T)	β^0^/β^0^	1
	IVS1-1G>A + IVS1-6 (T>C)/IVS1-6 (T>C)		1
	IVS1-6 (T>C)/IVS1-6 (T>C) + CD39 (C>T)/CD39 (C>T)	β^0^/β^0^	4
	IVS1-6 (T>C)/IVS1-6 (T>C) + CD39 (C>T)	β^+^/β^+^ or β^+^/β^0^	2
	IVS1-6 (T>C) + CD39 (C>T)/CD39 (C>T)	β^0^/β^0^	3
**Sub-total**			**20**
Compound heterozygous mutations	IVS1-1 (G>A)/uncharacterized	β^0^/β^0^ or β^0^/β^+^	4
	IVS1-6 (T>C)/uncharacterized	β^+^/β^+^ or β^+^/β^0^	4
	CD39 (C>T)/uncharacterized	β^0^/β^0^ or β^0^/β^+^	3
**Sub-total**			**11**
Other mutations	uncharacterized/uncharacterized	β^0^/β^0^ or β^+^/β^+^ or β^+^/β^0^	**31**
**Total**			100

**Table 4:** Distribution of hematological data, CAT and MDA for each group of genotypes among 100 Iraqi Thalassemia Patients.

Genotypes	N	RBC	HGB	HCT	MCV	MCH	Platelet Count	WB.10	LYM	NEUT	Ferritin	CAT	MDA	Number of Transfusions
Simple homozygous mutations	38	3.186 ± 0.456	7.89 ± 0.827	23.7 ± 2.313	4.329 ± 0.061	25.015 ± 2.008	461.157 ± 315.530	7.981 ± 3.879	45.494 ± 13.154	47.826 ± 10.629	2417.657 ± 2088.750	0.723 ± 0.346	3.516 ± 1.256	360.84 ± 154.956
Combined HBB mutations	20	3.10 ± 0.522	7.790 ± 0.877	23.120 ± 2.711	4.343 ± 0.078	25.410 ± 2.496	388.150 ± 202.676	8.150 ± 2.578	40.915 ± 13.092	49.605 ± 13.479	3731.5 ± 3154	0.818 ± 0.317	3.970 ± 1.648	395.5 ± 145.188
Compound heterozygous mutations	11	2.962 ± 0.450	7.345 ± 1.122	22.10 ± 3.336	4.324 ± 0.053	24.836 ± 1.825	330.727 ± 201.298	8.80 ± 4.509	45.027 ± 13.086	48.463 ± 11.033	2758.818 ± 2529.696	0.809 ± 0.450	4.073 ± 1.572	396 ± 119.157
Other mutations	31	3.150 ± 0.410	8.029 ± 0.751	24.251 ± 2.494	4.341 ± 0.102	25.625 ± 2.014	654.709 ± 942.351	9.825 ± 5.542	43.093 ± 12.967	49.216 ± 12.352	2972.806 ± 2589.370	0.852 ± 0.304	3.819 ± 1.418	414.97 ± 332.278

N: number; RBC: Red Blood Cell, HGB: Hemoglobin, HCT: Hematocrit, MCV: Mean Corpuscular Volume, MCH: Mean Corpuscular Hemoglobin, WBC.10: White Blood Cells (×10^3^/μL), LYM: Lymphocytes, NEUT: Neutrophils, CAT: Catalase, MDA: Malondialdehyde level.

**Table 5:** Association of *CAT* gene polymorphism: rs1001179 C>T and rs7943316 A>T in beta-thalassemia.

rs1001179 C>T	Genotype	Controls (n = 50) n (%)	Patients (n = 100) n (%)	OR (IC 95%)	AIC	*p* Value
Model						
Codominant	C/C	40 (80)	58 (58)			
	C/T	7 (14)	33 (33)	3.25 [1.31–8.07]	189.1	**0.019**
	T/T	3 (06)	9 (09)	2.07 [0.53–8.12]		
Dominant	C/C vs. C/T + T/T			2.90 [1.30–6.44]	187.4	**0.006**
Recessive	C/C+C/T vs. T/T			1.55 [0.40–6]	194.5	0.513
	Alleles					
	C	87 (87)	149 (74.5)			**0.016**
	T	13 (13)	51 (25.5)			
rs7943316 A>T						
Model						
Codominant	A/A	34 (68)	71 (71)			
	A/T	11 (22)	12 (12)	0.52 [0.21–1.30]	193.7	0.192
	T/T	5 (10)	17 (17)	1.63 [0.55–4.78]		
Dominant	AA vs. A/T + T/T			0.87 [0.42–1.81]	194.8	0.7063
Recessive	A/A + A/T vs. T/T			1.84 [0.64–5.33]	193.6	0.240
	Alleles					
	A	79 (79)	154 (77)			0.695
	T	21 (21)	46 (23)			

n: number OR: odds ratio; CI: confidence interval. For the rs1001179 C>T and rs7943316 A>T polymorphisms (which are not in the Hardy–Weinberg equilibrium), the Cochran Armitage test was used; bold: statistically significant *p*-values.

## Data Availability

The data presented in this study are available on request from the corresponding author due to privacy restrictions (the data are not publicly available).

## Supplementary Materials

The following supporting information can be downloaded at https://www.mdpi.com/article/10.3390/genes17080906/s1, Table S1: *CAT* Gene ARMS-PCR Details (rs1001179C>T, rs7943316 A>T); Table S2: SSP-PCR Details for the genotyping of HBB mutations (CD39 (C>T), IVS1-1 (G<A), and IVS1-6 (T>C); Table S3: Multivariate linear regressions between HBB mutation groups and hematological parameters; Table S4: β thalassemia studied mutation frequencies in Iraq and others countries [79,80,81,82,83,84,85,86,87,88,89,90].

## Abbreviations

The following abbreviations are used in this manuscript:

TDM	Transfusion-dependent β-thalassemia major
CBC	Complete blood count
CAT	Catalase
MDA	Malondialdehyde
RBC	Red Blood Cell
WBC	White Blood Cell
HGB	Hemoglobin
MCV	Mean Corpuscular Volume
MCH	Mean Corpuscular Hemoglobin
MCHC	Mean Corpuscular Hemoglobin Concentration
HCT	Hematocrit
TC	Total Cholesterol
TG	Triglycerides
HDL	High-Density Lipoprotein
LDL	Low-Density Lipoprotein
ARMS-PCR	Amplification Refractory Mutation System Polymerase Chain Reaction
SSP-PCR	Sequence Specific Primers Polymerase Chain Reaction
PCA	Principal Component Analysis
HWE	Hardy–Weinberg Equilibrium
