# Supplementary material for: Beta-Globin (HBB) Mutations and Catalase Gene Polymorphisms in Beta-Thalassemia Major Patients in Al-Diwaniyah, Iraq
Source: Genes (Basel). 2026 Jul 31;17(8):906. doi: 10.3390/genes17080906 (PMC13511826; doi:10.3390/genes17080906)
Supplement: Supplementary file 1 [file genes-17-00906-s001.zip › genes-4407400-supplementary.pdf]

Table S1. CAT Gene ARMS-PCR Details (rs1001179 C>T, rs7943316 A>T)

Component	Details	Product size
Primers (5'-3')	rs1001179 C>T: WT-F (C): GCCCTGGGTTCGGCTATC<br>Mut-F (T): GCCCTGGGTTCGGCTATT<br>Common-R: GAGGACTGCCTTCTGATTG	202 bp
	rs7943316 A>T: WT-F (A): AGCCTGAAGTCGCCACGAA<br>Mut-F (T): AGCCTGAAGTCGCCACGAT<br>Common-R: GCTTTCTAAACGGACCTTC	220 bp
Reaction Mix (25 μL)	DNA (5 μL), allele-specific primer (2 μL, 10 pmol), common primer (2 μL, 10 pmol), GoTaq ® G2 Green Master Mix (Promega) (12.5 μL), PCR water (3.5 μL)	
PCR Cycling (Bio-Rad thermocycler)	Initial: 95°C/5 min; 35 cycles (95°C/30 s, 55°C/30 s, 72°C/30 s); Final: 72°C/5 min	

Table S2. SSP-PCR Details for the genotyping of HBB mutations (CD39 (C>T), IVS1-1 (G<A), and IVS1-6 (T>C)

Component	Details	Product size	Reference
Primers (5'-3')	CD39 (C>T) mutant : GACTCAAAGAACCTCTA	439 bp	[40] Gomes et al., 2017
	IVS1-1 (G<A)mutant : GTAACCTTGATACCAAA	268pb	
	IVS1-6 (T<C)mutant : GTCTTGTAACCTTGATG	273pb	
	Internal control<br>F: GGCTGTCATCACTTAGACCTCA<br>R: AGAAGGGGAAAGAAAACATCAA	659pb	
Reaction Mix (25 μL)	Genomic DNA: 5 μL<br>Mutant primers: 1 μL each (10 pmol/μL)<br>Internal F+R: 1 μL each<br>GoTaq® G2 Green Master Mix (Promega): 12.5 μL<br>H_2O: 2.5 μL		
PCR Cycling (Bio-Rad)	95°C/5 min<br>35 cycles: 95°C30 s, 55°C/30 s, 72°C/30 s<br>72°C/5 min		

Table S3: Multivariate linear regressions between HBB mutation groups and hematological parameters

Covariate	Coefficient (B)	Standard error	Standardized β	95% CI	P value
Constant	1.048	0.144	-	0.762 to 1.335	<0.001
Age	-0.013	0.005	-0.205	-0.023 to -0.003	0.013*
Gender	-0.010	0.068	-0.014	-0.144 to 0.125	0.888
β^+ mutation	-0.052	0.113	-0.046	-0.277 to 0.172	0.646
β^0 mutation	-0.154	0.076	-0.205	-0.305 to -0.003	0.046*

Dependent variable: CAT; sample size: N = 100; covariates: age and gender; predictors of interest: β+ mutation and β0 mutation; $R^2 = 0.097$; adjusted $R^2 = 0.059$; $F(4,95) = 2.563$; model $p = 0.043$.

Table S4. β thalassemia studied mutation frequencies in Iraq and others countries

Country			IVSI-1 (G>A) (%)	IVS1-6 (T>C) (%)	CD39 (C>T) (%)	No.of chromosomes	References
Iraq	Iraqi Arabs	Al-Diwanyah	29	25.5	25.5	200	Current study
		Karbala	6.67	4.17	4.17	120	[19] Al-Fatlawi et al.,2024
		Baghdad	2	24	0.5	204	[66] Al-Allawi et al.,2015
	Iraqi Kurds	Erbil	3.6	33.1	1.2	166	[67] Shamoon et al.,2015
		Duhok	3.9	32.4	2	102	[68] Al-Allawi *et al.*,2014
		Kirkuk	2.5	3.75	2.5	100	[19] Al-Fatlawi et al.,2024
	North Iraq	Nineveh	6.4	9.6	7.4	188	[69] Eissa *et al.*,2015
Other countries	Syria		9.2	1.4	21.3	192	[21] Kocak and Balasar,(2024)
	Iran		2.68	1.54	1.34	63 468	[70] Mahdieh and Rabbani ,2016
	Azerbaijan		2.06	4.18	1.76	240	[79] Kuliev et al.,1994
	Turkey		6.88	7.10	8.63	626	[80] Karaer et al.,2023
	Jordan		10	8.3	4.6	244	[81] Sadiq *et al.*,2001
	Saudi		5.8	4.4	20.3	138	[82] Al-Ali *et al*.,2005
	Kuwait		5.74	7.38	5.74	36	[83] Adekile *et al.*,2005
	Qatar		1.54	NA	1.54	62	[84] Al-Obaidli *et al.*,2007
	Bahrain		3	NA	24.2	66	[85] Jassim *et al*,1998
	Oman		1.4	0.12	1.17	198	[86] Daar *et al.*,1998
	Lebanon		4.8	40.4	NA	146	[87] Qatanani *et al.*,2000
	Sudan		16	28	NA	200	[88] ElShilkh *et al.*,2019
	Tunisia		3.31	10.5	48.76	121	[89] Haj Khelil A et *al.*,2004
	Pakistan		1.7	NA	0.2	602	[90] Khan and Riazuddin 1998
	Brazil		0	18	22	50	[40] Gomes *et al.*,2017
	Highest frequency based on HbVar		Spanish (31.79%); Hungarian (28.13%); Syria (17%); Lebanese (15%); Greek (13.65%)	Macedonian (18.56%); Azerbaijan (17.3%); Sicilian (16%); Turkish (14.75%)	Sardinian (95.73%); Italian (66.84%); Argentine (47.06%); French (41.9%)		[70] Mahdieh and Rabbani (2016)

NA: Not available
